# Novel Research Models for *Staphylococcus aureus* Small Colony Variants (SCV) Development: Co-pathogenesis and Growth Rate

**DOI:** 10.3389/fmicb.2020.00321

**Published:** 2020-02-28

**Authors:** James Lee, Peter S. Zilm, Stephen P. Kidd

**Affiliations:** ^1^Department of Molecular and Biomedical Science, The University of Adelaide, Adelaide, SA, Australia; ^2^Research Centre for Infectious Diseases, Adelaide, SA, Australia; ^3^Australian Centre for Antimicrobial Resistance Ecology, Adelaide, SA, Australia; ^4^Faculty of Health and Medical Sciences, The University of Adelaide, Adelaide, SA, Australia

**Keywords:** *Staphylococcus aureus*, small colony variants, continuous culture, prolonged growth, co-colonization

## Abstract

*Staphylococcus aureus* remains a great burden on the healthcare system. Despite prescribed treatments often seemingly to be successful, *S. aureus* can survive and cause a relapsing infection which cannot be cleared. These infections are in part due to quasi-dormant sub-population which is tolerant to antibiotics and able to evade the host immune response. These include Small Colony Variants (SCVs). Because SCVs readily revert to non-SCV cell types under laboratory conditions, the characterization of SCVs has been problematic. This mini-review covers the phenotypic and genetic changes in stable SCVs including the selection of SCVs by and interactions with other bacterial species.

## Introduction

*Staphylococcus aureus* is a Gram positive, facultative anaerobe and non-motile opportunistic bacterium. Approximately 30% of the human population are asymptomatic carriers and 60% are intermittent carriers. The anterior nares serve as the major reservoir and thereby becomes a source for systemic infections (Tong et al., 2015) as *S. aureus* is also an important human pathogen responsible for morbidity and mortality worldwide (Tong et al., 2015; Kahl et al., 2016). These infections can be difficult to clear due to a persistent reservoir of *S. aureus* which survives antibiotic treatment.

Even within a genetically clonal population, it is now recognized that there exists a variety of phenotypes which can be referred to as alternative lifestyles (Balaban et al., 2004; Wood et al., 2013). In contrast to classical resistance mechanisms, these phenotypes confer tolerance to antimicrobials, where there is limited or no growth, but not cell death (Keren et al., 2004). The alternative lifestyles arise through disruptions to cellular activities and not through the acquisition of new virulence genes (Balaban et al., 2004; Wood et al., 2013). These lifestyles involve forming quasi-dormant sub-populations during infection which have increased fitness in unfavorable conditions. While the mechanisms may differ, these sub-populations generally create a reservoir of *S. aureus* within an anatomical niche which are difficult to clear by the host immune response or therapeutic means and can revert to their parental, active cell type (Keren et al., 2004; Singh et al., 2009). These phenotypic switches in *S. aureus* include formation of biofilms, persister cells and Small Colony Variants (SCVs).

### *S. aureus* Small Colony Variants

*Staphylococcus aureus* SCVs are characterized by small colony size, impeded growth, loss of carotenoid pigment (Proctor et al., 2006; Melter and Radojevič, 2010) non-hemolytic and coagulase negative (Thomas, 1955; Quie, 1969; Melter and Radojevič, 2010; Bui et al., 2015). SCVs exhibit changes in structural morphology and dysfunction in cell separation (Kahl et al., 2003a). While they have been extensively studied there remain unknown nuances to their development and reversion. It is known that they form through auxotrophy in elements of the electron transport chain and ATP production (Kahl et al., 2003a; Proctor et al., 2006). These are either single or a combination of auxotrophy in the biosynthesis of menadione, hemin or thymidine (Kahl et al., 2003a; Kohler et al., 2008; Melter and Radojevič, 2010; Maduka-Ezeh et al., 2012; Dean et al., 2014; Horiuchi et al., 2015) CO_2_ (Thomas, 1955) and fatty acids (Schleimer et al., 2018). Many SCV isolates have no defined auxotrophism (Edwards, 2012) and various mutations in the electron transport chain that result in SCV (Proctor, 2019) are not observed in clinical isolates.

Although complex, reduced ATP production is associated with variations in cell-wall biosynthesis and carotenoid pigment (Proctor et al., 2006, 2014). Global genetic change has been observed in the switch to SCVs, with increased biofilm formation (Singh et al., 2010), autolysis (Bui et al., 2015), expression of adhesive proteins (Mirani et al., 2015) and decreased expression of secreted virulence factors (Melter and Radojevič, 2010; Tuchscherr et al., 2010; Ou et al., 2016).

Small Colony Variants persist intracellularly, avoiding clearance by the host immune response (Melter and Radojevič, 2010; Kahl et al., 2016). Examples of SCV infections include the lungs in patients with cystic fibrosis (Kahl et al., 1998, 2003b; Schwerdt et al., 2018), bovine mastitis (Atalla et al., 2011), osteomyelitis (Proctor et al., 1995), and foreign body infections (Baddour and Christensen, 1987; Von Eiff et al., 1999) including prosthetic-periprosthetic joint infection (PJI) (Yang et al., 2018). SCVs have an innate tolerance of antibiotics which is not associated with resistance genes (Edwards, 2012). The lack of an electrochemical gradient in their cell wall prevents aminoglycosides from entering the cell and the growth dormancy reduces the effectiveness of antibiotics that target metabolic processes of actively growing cells (Proctor et al., 1998; Melter and Radojevič, 2010; Garcia et al., 2013). This allows selection of SCV and chronic infection in antibiotic treatment, such as the use of gentamicin in bone cement for prosthetic joint implants (Chang et al., 2013). SCVs also have greater intracellular uptake and survival within non-professional phagocytic cells (fibroblast, epithelial, endothelial, osteoblast, osteocytes, keratinocytes) and professional phagocytes (Garzoni and Kelley, 2009). *In vitro* cell culture models of non-professional phagocytic cells shows a dependence on fibronectin binding proteins (FbNPs) for adhesion and activating cytoskeletal remodeling (Sendi and Proctor, 2009). Many surface adhesion molecules are upregulated in SCVs (Gómez-González et al., 2010; Bui and Kidd, 2015). Down regulation of α-toxin is required to keep the host cell alive and allow persistence intracellularly (Garzoni and Kelley, 2009; Sendi and Proctor, 2009).

Clinically isolated SCVs are often not stable and revert to their non-SCV cell type when cultured in the laboratory (Edwards, 2012; Kriegeskorte et al., 2014a; Bui et al., 2015). Also, clinical isolates of SCVs from diseased tissues give no indication of the parental type, leaving questions as to the genetic and molecular mechanisms involved during the transition from the original cell to its SCV phenotype. While SCVs can be abundant in persistent infections (Bates et al., 2003), identifying and culturing clinical samples is indeed difficult. The use of genetically stable SCVs (sSCV) has greatly improved our understanding of SCVs (Kriegeskorte et al., 2014a; Bui et al., 2015). Models of sSCV *S. aureus* include mutants auxotrophic to menadione (Schaaff et al., 2003; Lannergård et al., 2008; Dean et al., 2014; Pader et al., 2014), hemin (Balwit et al., 1994; Von Eiff et al., 1997; Schaaff et al., 2003), thymidine (Balwit et al., 1994; Von Eiff et al., 1997; Besier et al., 2007; Chatterjee et al., 2008; Kriegeskorte et al., 2014a; Kittinger et al., 2019), fatty acids (Bazaid et al., 2018; Schleimer et al., 2018) CO_2_ (Thomas, 1955; Gómez-González et al., 2010), chorismite synthesis (precursor for aromatic amino acids and menaquinone biosynthesis) (Zhang et al., 2017), selection in gentamicin (Balwit et al., 1994) and serial passage in mice models immunized against capsular polysaccharide (Tuchscherr et al., 2008).

### Induction of Stable SCV by Prolonged Slow Growth Under Nutrient Limitation

Alternative methods of inducing the transition to SCV and formation of sSCV have been reported. Bui et al., grew the clinical blood isolate, WCH-SK2 for a prolonged time-period by continuous culture under nutrient limiting conditions and a low growth rate using an *in vitro* system allows single parameters, outside the complexity of *in vivo* conditions (Bui et al., 2015). Introduced oxidative stress and growth over 209 generations (60 days) produced a sSCV which dominated the population. Accumulated oxidative stress causes damage to DNA, and the SOS response creates mismatch repairs and increased rate of mutation. Previous studies have shown the frequency of SCV formation increases with the rate of mutation (Schaaff et al., 2003; Vestergaard et al., 2015; Lacoma et al., 2019).

This methodology has been replicated to select for sSCV in other *S. aureus* strains; such as MW2, community acquired MRSA blood isolate (Lee et al., unpublished). Unlike sSCV created in the laboratory (by site directed mutations of specific genes), continuous culture considers the phenotypic and genetic responses in a time-dependent manner within nutrient limiting conditions. This enables one strain to be observed in transition from a parental population to one with a diversity of cell types and then dominated by SCVs.

Whole genome sequencing of WCH-SK2 and WCH-SK2-SCV revealed 24 genetic events; single nucleotide polymorphisms (SNPs) or insertions-deletions (indels) which could be implicated with the switch to a sSCV while under nutrient starved, stressed conditions (Bui et al., 2015; Table 1). The instability of some clinical SCV isolates suggests their phenotype is transcriptionally controlled (and/or post-transcription) rather than by stable SNPs. The *in vivo* environment with combined stressors likely pressures *S. aureus* to become intracellular and remain as a SCV.

**TABLE 1:** The switch to a Small Colony Variant (SCV) by WCH-SK2 through continuous culture was associated with genetic events (SNPs) in global regulators of virulence.

	**Agr**	**MgrA**	**ArlRS**	**SigB**
**Extracellular components**				
*cap5A-P*	+	+	+	+
*icaABCD*		+	+	
*ebh*				
*clfA*				+
*fnbA*	−			+
*fnbB*	−			+
*SpA*	−	−	−	+
**Leukocidins**				
*lukDE*	+	+	−	−
*lukSF*	+	+		−
*lukM*		+		−
**Exotoxins**				
*hla*	+	+		−
*hlb*	+	+		−
*seb*	+	+		−
*sea, seb, sec*	+			
*etaA, etaB*	+			
*tst-1*	+			
**Secretory proteases**				
*splA, splB, splC, splD*	+	+	−	−
*sspA*	+	+		−
*sspBC*	+		−	−
*nuc1*	+	+		−
*srtA*		+		−
**Autolysis**				
*lytM*		−		+
*lytN*		−	−	
*lytSR*			+	
*atl*		−		
*cidA*		−		
*lgrAB*		+	+	−
**Metabolism**				
Lactose metabolism		−	−	
Urea metabolism		−	−	
Arginine deaminase		−	−	

*Shown are the change in expression of genes regulated in *S. aureus* pathogenesis through these global regulators. +, increased expression, −, decreased expression.*

### MgrA –A Global Regulator of Virulence

The transition of WCH-SK2 to an sSCV was suggested to be associated with a SNP in the DNA binding domain of *mgrA* (R92C change in MgrA). This mutation could impact the binding kinetics (protein-DNA interaction); previous mutations studied in MgrA suggests mutations at this point would make MgrA non-functional. The loss of *mgrA* function has not been previously reported in any SCV clinical isolates, but present a new perspective for sSCVs. The global regulator MgrA is known to function downstream to the two-component ArlRS (Crosby et al., 2016; Kwiecinski et al., 2019) and controls genes including the upregulation of capsular polysaccharide, α-toxin, leukocidins, coagulase and protein A (Luong et al., 2006; Lei et al., 2019), all of which are virulence factors downregulated in SCVs (Proctor et al., 2014; Bui and Kidd, 2015). The loss of *mgrA* enhances autolysis (Ingavale et al., 2003, 2005), invasion of HeLa cells (Lei et al., 2019), increases biofilm formation (Trotonda et al., 2008; Crosby et al., 2016) and increases expression of microbial surface components recognizing adhesive matrix molecules (MSCRAMM) such as Ebh, a large 1.1-MDa protein (Crosby et al., 2016) and indeed other surface proteins (Kwiecinski et al., 2019). While the loss of function of MgrA has not been characterized in the context of formation or stability of SCVs, the downregulation of other homologous SarA family proteins have been associated with the formation of SCV (Kahl et al., 2005; Kriegeskorte et al., 2014b; Mirani et al., 2015).

### Agr – The Quorum Sensing System

Another major determinant for the production of toxins and extracellular enzymes is the quorum sensing two component system (TCS), Agr. Quorum sensing is controlled by secretion of the *agr* inducing peptide (AIP) to upregulate *agr* expression in surrounding *S. aureus*. The downregulation or loss of *agr* has previously been reported in clinical SCVs (Vaudaux et al., 2002; Kahl et al., 2005; Moisan et al., 2006; Kriegeskorte et al., 2014b). RNAIII is the effector molecule of the Agr TCS and controls the upregulation of secreted proteins and toxins and downregulation of cell surface proteins (Rescei et al., 1986; Abdelnour et al., 1993; Arvidson and Tegmark, 2001; Novick, 2003; Cheung et al., 2004). SCVs can display changes in RNAIII in persistent infections through the RNA degrasome, production of small RNAs and toxin-antitoxins (Proctor et al., 2014).

### RsbU – A Regulator of SigB

In stressful environments, *S. aureus* employs alternative sigma factor B (SigB) to sense changes in the environment and alter its gene expression profile accordingly. The SigB expression profile acts in opposition to the Agr TCS where there is an increased expression of cell surface proteins for colonization and decreased expression of secreted proteins and toxins (Kullik et al., 1998; Bischoff et al., 2004; Jonsson et al., 2004). RsbU positively regulates *sigB* in a growth phase dependent manner, with SigB expression highest during late exponential phase (Senn et al., 2005). SigB can regulate the switch to dormancy in *S. aureus* in opposition to *agr* and its regulation of virulence. High levels of SigB have been found in clinical SCVs isolated from cystic fibrosis (Moisan et al., 2006; Mitchell et al., 2008, 2013), osteomyelitis (Tuchscherr et al., 2017) and bovine mastitis (Mitchell et al., 2010a). SigB activity is associated with downregulation of the *agr* system (Yarwood and Schlievert, 2003) and immunogenic virulence factors (enterotoxins, hemolysins, secreted proteases) and shown to allow SCVs to persist intracellularly within human endothelium (Tuchscherr et al., 2015). This also is associated with the increase in expression of FnBPs such as FnbA which then contributes to biofilm formation, adhesion and intracellular persistence. Furthermore, *sigB* expression is required for intracellular replication of SCVs, and was shown to confer greater fitness in a pulmonary mouse model (Mitchell et al., 2013). SigB and subsequent *agr* repression is required for formation of SCVs in response to aminoglycoside stress (Mitchell et al., 2010a).

## Selection of SCV Within Polymicrobial Environments

### Presence of *Pseudomonas aeruginosa* Selects for SCV

The diverse population of bacteria within the human microbiome means *S. aureus* is rarely in isolation during infection or commensal carriage. The specific nature of the microbial population within a niche creates a vast array of complex interactions between *S. aureus* and other bacterial species and this affects its ability to colonize, acquire nutrients and proliferate. The local bacteria are known to impact on *S. aureus* cell types. One such interaction is a co-culture of *S. aureus* and *P. aeruginosa* which has been found to select for SCV or persisters of *S. aureus*. Various *S. aureus* infections are frequently isolated alongside *P. aeruginosa* such as in soft tissue infections, diabetic foot infections, osteomyelitis and within cystic fibrosis airways (Kahl et al., 2016). These two species can act competitively or cooperatively, such as *P. aeruginosa* secretions of LasB; an elastase which removes lung surfactant and prevents uptake by macrophages to allow effective colonization and persistence in the lung (Hotterbeekx et al., 2017).

Conversely, the secretion of an antistaphyloccocal metabolite, 4-hydroxy-2-heptylquinoline-N-oxide (HQNQ), by *P. aeruginosa* inhibits *S. aureus* growth through interrupting its electron transport chain and ATP production (Machan et al., 1992; Proctor, 2019). Long term exposure to physiological concentrations of HQNQ in combination with aminoglycosides *in vitro* resulted in high proportions of menadione SCVs (Hoffman et al., 2006). Indeed, in the co-existence of these bacteria, *S. aureus* becomes less susceptible to vancomycin and other antibiotics (Orazi and O’Toole, 2017; Radlinski et al., 2017). This has also been recognized through clinically relevant analyses (Mitchell et al., 2010b; Fugère et al., 2014).

No other interaction between *S. aureus* and other bacterial species has been found to directly induce SCV formation. However, if we consider the selective pressures which allow SCV to survive and dominate a population of cells through increased fitness over their parental cell types; including a reduced nutrient availability, antibiotics, phagocytosis, extreme pH; we can deduce that the interactions between *S. aureus* and other bacterial species which negatively affect *S. aureus* pathogenesis and survival may also be selecting for SCV (or indeed, other quasi-dormant cell types). *Corynebacterium* spp. and other Staphylococci are known to inhibit *S. aureus* virulence (in particular, through blocking *agr* function), nutrient acquisition and adhesion (Iwase et al., 2010; Wollenberg et al., 2014; Ramsey et al., 2016). *Streptococcus* spp. and *Staphylococcus lugdunensis* produce exoproducts which actively kill *S. aureus* (Zipperer et al., 2016; Wu et al., 2019). Many of these species we describe later are carried in the nares (Lina et al., 2003; Huttenhower et al., 2012), a potentially ideal anatomical niche for selecting and forming SCVs. Thymidine auxotroph SCVs have been isolated from the nares in a patient with AIDS (von Eiff et al., 2004) and pulmonary fibrosis (Cleeve et al., 2006). There are cases of *S. aureus* progressing from nasal colonization to bacteremia without the acquisition of additional virulence genes and SNPs in *arlS* and *agrA* (Benoit et al., 2018); genetic profiles previously reported in clinical SCVs (Kahl et al., 2005; Kohler et al., 2008; Kriegeskorte et al., 2014b). However, in contrast, *S. aureus* nasal colonization has been shown to favor dispersed cell-types rather than biofilm formation (Krismer and Peschel, 2011) which may imply SCV are less fit in the nares. The interactions between *S. aureus* and other bacterial species has not been researched in detail, and so we review the interactions in the context of selection of SCV.

*Staphylococcus epidermidis* is commonly found to out-compete *S. aureus* in the nares (Lina et al., 2003; Frank et al., 2010; Lee et al., 2019). *S. epidermidis* can block *agr* quorum sensing in *S. aureus* (Otto et al., 1999) and production of the serine protease, Esp, which inhibits *S. aureus* colonization through inhibiting biofilm formation and synergistically increasing the ability of Human-beta defensin 2 to clear *S. aureus* (Iwase et al., 2010). In a similar fashion, *Staphylococcus caprae*, a skin commensal, also interferes with *S. aureus* colonization and produces an AIP which blocks *S. aureus agr* sensing (Paharik et al., 2017).

*Staphylococcus lugdunensis* produces a peptide antibiotic, lugdunin, which has bactericidal effects against *S. aureus* (Zipperer et al., 2016). The mechanism of action of lugdunin against *S. aureus* has not been determined, and so whether this bactericidal effect selects for SCV is unclear. However, it has been shown to act synergistically with the innate immune response, where lugdunin increases recruitment of monocytes and neutrophils to keratinocytes (Bitschar et al., 2019).

*Propionibacterium* spp. produces an exoproduct coproporphyrin III (CIII) which induces aggregation and biofilm formation in *S. aureus* within acidic conditions (pH 4–6) (Wollenberg et al., 2014). SarA was shown to be involved in CIII mediated biofilm, however, the role of other biofilm regulators was not tested in this study.

*Streptococcus pneumoniae* production of hydrogen peroxide is able to kill *S. aureus* within *in vitro* conditions (Uehara et al., 2001; Regev-Yochay et al., 2006; Wu et al., 2019). Hydrogen peroxide can oxidize iron groups to damage proteins, or generate OH^–^ to cause DNA damage (Keyer and Imlay, 1996). However, a study using the nasal cavities of neonatal rats showed this hydrogen peroxide is not enough to affect *S. aureus* colonization (Margolis, 2009). It is known that over prolonged periods of time *S. aureus* in its SCV state can tolerate hydrogen peroxide (Painter et al., 2015) and so *S. aureus* may switch to a SCV when assaulted by *S. pneumoniae* generated hydrogen peroxide.

Both *S. aureus* and *Cornyebacterium* spp. are common nasal colonizers (Frank et al., 2010) and *in vitro* co-colonization of *S. aureus* with *Corynebacterium* spp. results in a shift of *S. aureus* from a virulent to a commensal state, with a strongly inhibited *agr* (Ramsey et al., 2016). Co-cultures with *Corynebacterium* spp. (including nasal strains *C. striatum*, *C. amycolatum*, *C. accolens*, *C. pseudodiptheriticum* and a soil strain *C. glutamicum*) interferes with AIP-1 (*agr* activator molecule) and increases clearance of *S. aureus* within a murine model of infection (Ramsey et al., 2016). This co-culture is associated with a global change in gene regulation, with a notably increased expression of *spa* (260-fold) and down-regulation of *agr*. The loss of *agr* function has been associated with resistance to the bacteriocidal effects of *C. pseudodiptheriticum* (Hardy et al., 2019), however, the bacteriocidal mechanism by *C. pseudodiptheriticum* has not been established. The loss of *agr* activity and increased expression of adhesive surface proteins are common characteristics of SCV and this is an indicator that *S. aureus* may resist clearance by *Corynebacterium* spp. by switching to SCV.

## Conclusion

To advance both diagnosis and treatment protocols for *S. aureus* infections, it is vital to understand the molecular mechanisms that select for or induce the formation of SCVs. Current therapeutic treatments are becoming less effective against persistent *S. aureus* infections. Given the tendency for SCVs to revert, and the difficulty in their culturing, means research into *S. aureus* SCVs still remains a challenge. The use of continuous culture to select for SCVs amongst a population of cells has a great potential in discovering the molecular mechanisms involved in the transition into a SCV state which other sSCV models cannot clearly define.

Another aspect of SCV formation to consider is the influence of the microbiota co-existing with *S. aureus*. Studies with *S. aureus* and *P. aeruginosa* have shown this interaction selects for quasi-dormant cell types. The conditions in the nares, interactions with other bacterial species and their ability to persist intracellularly may favor the formation of SCVs. These can then transit around the human body and evade the host immune response. This provides a new perspective on the nasal carriage of *S. aureus* and the increased risk of endogenous *S. aureus* infection (Von Eiff et al., 2001; Kluytmans and Wertheim, 2005; Stanaway et al., 2007; Haleem et al., 2014; Dunyach-Remy et al., 2017). This includes immunocompromised sites such as the diabetic foot (Lavery et al., 2006) or a foreign body surface for attachment and biofilm production (Kahl et al., 2016; Yang et al., 2018).

## Author Contributions

JL, PZ, and SK each contributed to the design, construction, and writing of this manuscript.

## Conflict of Interest

The authors declare that the research was conducted in the absence of any commercial or financial relationships that could be construed as a potential conflict of interest.
